# Application and Research Status of Long-Wavelength Fluorescent Carbon Dots

**DOI:** 10.3390/molecules28227473

**Published:** 2023-11-08

**Authors:** Yujia Cheng, Guang Yu

**Affiliations:** Mechanical and Electrical Engineering Institute, Zhongshan Institute, University of Electronic Science and Technology of China, Zhongshan 528400, China; chengyujia@zsc.edu.cn

**Keywords:** carbon dots, fluorescent nanomaterial, versatile applications

## Abstract

This article discusses the application and research status of long-wavelength fluorescent carbon dots. Currently, there are two main methods for synthesising carbon dots (CDs), either from top to bottom, according to the bulk material, or from bottom to top, according to the small molecules. In previous research, mainly graphite and carbon fibres were used as raw materials with which to prepare CDs, using methods such as arc discharge, laser corrosion, and electrochemistry. These preparation methods have low quantum efficiencies and afford CDs that are limited to blue short-wavelength light emissions. With advancing research, the raw materials used for CD preparation have expanded from graphite to biomaterials, such as strawberry, lime juice, and silkworm chrysalis, and carbon-based molecules, such as citric acid, urea, and ethylenediamine (EDA). The preparation of CDs using carbon-based materials is more rapid and convenient because it involves the use of microwaves, ultrasonication, and hydrothermal techniques. Research on developing methods through which to prepare CDs has made great progress. The current research in this regard is focused on the synthesis of CDs, including long-wavelength fluorescent CDs, with a broader range of applications.

## 1. Introduction

**Figure 1:**
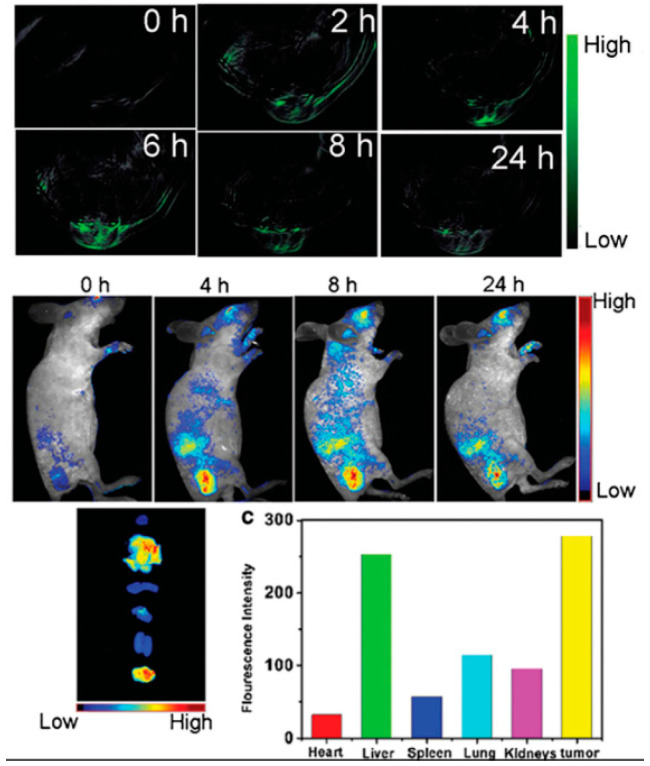
Carbon dots utilised for multimodal imaging in mice [13].

Carbon dots (CDs) are fluorescent nanomaterials that are smaller than 10 nm. The origin of these types of materials can be traced back to 2004, when Xu et al. [1] purified a single-walled carbon nanotube fragment using electrophoresis and isolated nanoparticles that exhibited fluorescence properties. In 2006, Sun et al. [2] used graphite and black charcoal as raw materials with which to synthesise nanoparticles with improved fluorescence properties. This led to the nomination of CDs as the first official carbon-based fluorescent nanoparticles. Compared with traditional fluorescent materials, such as organic dyestuff and quantum dots, CDs are simple to synthesise, have low consumption, and possess superior luminescence properties and biocompatibility [3,4]. In addition, the surfaces of CDs are enriched in water-soluble groups. CDs readily combine with inorganic molecules, organic molecules, and biomolecules, allowing them to exhibit multifunctionality. CDs are widely used in biological imaging, heavy metal detection, catalysis, and drug loading, making them a current research hotspot [5,6,7,8]. The research targets are divided into two aspects: improving the quantum yield (QY) and exploring the versatile applications of CDs [9,10]. Exploring the possible applications of CDs, Tian [11] developed full-colour CDs for use in white-light-emitting diodes (WLEDs). In 2017, Zhong [12] synthesised green CDs for Ag^+^ detection, using 1,2-phenylenediamine and formaldehyde as raw materials via hydrothermal synthesis. Additionally, CDs are widely used in catalytic reactions and electro-optical devices. For example, Wang synthesised silane-functionalised CDs, G-SiCDs, and R-SiCDs with green and red emissions, using a one-step solvothermal route. Initially, a red light-emitting diode (LED) was prepared by combining blue-fluorescence LED chips with R-SiCDs. Subsequently, the ratios of G-SiCDs and R-SiCDs were adjusted, and a WLED with a chrominance value of 88 was synthesised based on trichromatic fluorescence. The chrominance of the WLED was higher than that of the double-colour-based LED prepared using G-SiCDs by a value of 30. This led to an improvement in the ability of the WLED to present the true colour of the irradiated object. Ge [13] used polythiophene phenylpropionic acid as a raw material with which to prepare new-type red fluorescent CDs. The range of these CDs is 400–750 nm, and their maximum emission wavelength is 640 nm. The in vivo fluorescence imaging, which was conducted on mouse models, is shown in Figure 1. Under a near-infrared laser, the photoacoustic response was very strong, and photothermal conversion efficiency was high (η = 38.5%), proving that these CDs can be applied to the areas of multichannel fluorescence imaging, photoacoustic imaging, and photothermal therapy. Moreover, it is hopeful that these CDs have the potential to be used for cancer diagnosis and treatment. Zhong [14] used 1,2-paraphenylenediamine and carbofural as precursors. Nitrogen-doped orange florescence CDs (QE = 14.3%) were composed via 180 °C hydrothermal reaction for 3 h, and can be applied to Ag^+^ detection in water and to A549 human lung cancer cell imaging.

## 2. Summary of Previously Reported Synthetic Routes to CDs

The incomplete carbonisation of CD surfaces is advantageous because the existing interstitial structure makes the CD surface easy to modify. Research has shown that atom doping can improve the fluorescence properties of CDs [15,16,17,18].

### 2.1. Non-Metallic Element Doping

Early experiments suggested that nitrogen atom doping is the most effective, due to the similarity in radius between nitrogen and carbon atoms and the high electronegativity of nitrogen. Nitrogen atoms can provide electrons to carbon atoms, thus altering the internal structure and improving the luminescent properties of CDs. Qian et al. [19] used 1,2-ethylenediamine (EDA), 1,3-propylenediamine (PDA), 1,4-tetramethylenediamine, and ethylene glycol as dopants, and the different nitrogen contents in these materials had different effects on the luminescent properties of CDs. The QYs of these nitrogen-doped CDs were between 20.4% and 36.3%, far surpassing those of undoped CDs. Reckmeier et al. [20] utilised a hydrothermal method through which to prepare nitrogen-doped carbon quantum dots (CQDs), which exhibited a fluorescence quantum efficiency of nearly 80%.

Furthermore, the homology of carbon atoms and nearby elements, like Si, P, and S, can have an effect on doping. Guo et al. [21] utilised sodium citrate and L-cysteine as precursors in order to synthesise CDs with a quantum efficiency up to 68%. He et al. [22] used triethoxysilane and EDA as raw materials with which to prepare blue CDs with a quantum efficiency of 29.7%. These CDs were successfully utilised for the detection of ferric ions, demonstrating that doping with Si and N elements can enhance the fluorescence properties of CDs. Wang et al. [23] used maltose, hydrochloric acid, and phosphoric acid as raw materials, increasing the quantum efficiency of the tested CDs from 9.3% to 15%. The CDs also showed a linear response to ferric ions, making them a promising material for sensing applications. Two types of nitrogen–boron-doped CDs were prepared by Ye et al. [24] in order to detect 2,4,6-trinitrophenol in cells and Hg^2+^ in water. Yang et al. [25] used phytic and citric acids, which are rich in phosphorus, to prepare P-doped CDs, which were effective in the sensitive detection of copper ions.

### 2.2. Metallic Element Doping

Doping CDs with metal ions has been reported to significantly impact their properties and potential applications. Near-infrared (NIR) laser induction therapy is a unique, minimally invasive or non-invasive cancer treatment that has attracted considerable attention. Guo et al. [26] prepared excitation-dependent blue-fluorescent Cu-N-doped CDs using ethylenediaminetetraacetic acid (EDTA-2Na) and CuCl_2_ via one-step hydrothermal synthesis. Cooperative photothermal/photodynamic therapy has been found to substantially inhibit cancer. Moreover, Cu-N-CDs can function as a type of fluorescent probe and infrared thermal developer. In addition, the treatment process using Cu-N-CDs is non-invasive, making it a promising approach for application in biomedical imaging and therapy. Pakkath [27] employed citric acid as the carbon source and rapidly synthesised (within 6 min) CDs doped with ethylenediamine-functionalised transition-metal ions (Mn^2+^, Fe^2+^, Co^2+^, and Ni^2+^) via the microwave method. These CDs had a quantum efficiency of up to 50.84% and exhibited biocompatibility when used for fluorescence biological imaging of human colon cancer cells (SW480). He et al. [28] developed Gd-doped CDs with excellent magnetic resonance and an excellent cell imaging capability, as well as a high gene transfer efficiency and potent antiserum capacity. Even in an environment containing 10% serum, these CDs exhibited a gene transfer efficiency 74 times higher than that of polyethyleneimine (PEI, 25 kDa). Furthermore, they produced high permeability and carryover effects in solid tumours, resulting in enhanced imaging. Thus, metal ion-doped CDs are promising for medical applications—particularly as contrast media and targeting agents—owing to their unique properties.

**Figure 2:**
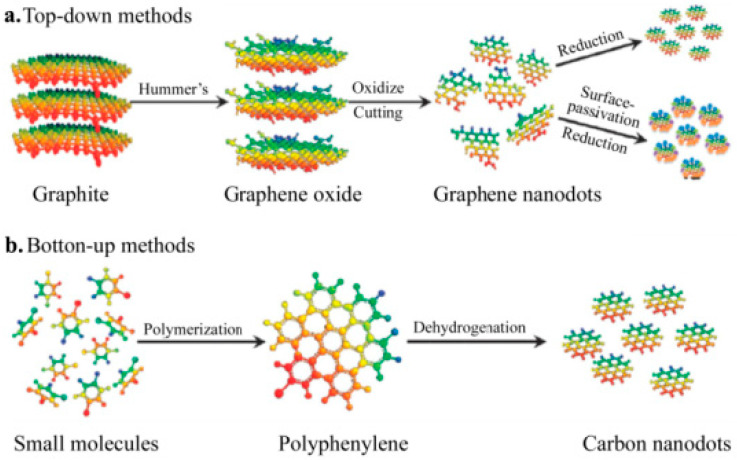
Schematic of the synthesis methods for carbon dots [28]: (**a**) ‘top-down’ method, (**b**) ‘bottom-up’ method.

CD preparation methods can be divided into two categories according to the mechanism used: top-to-bottom (or ‘top-down’) and bottom-to-top (or ‘bottom-up’) (Figure 2a). The quintessence of the top-down method is physical shearing or chemical stripping of carbon-rich fragments, such as graphene and carbon nanotubes (CNTs), into nanoscale CDs (graphene is shown as green, carbon nanotubes are shown in yellow). For instance, in a previous study, graphene was used as the raw material. A two-step shearing process was employed to produce monodispersed CDs from HNO_3_-octadecene amine hydrazine, and these CDs were then used for the preparation of white light-emitting diode (WLED) components. Lu used an electrochemical method to strip graphite in an ionic solution and produce blue-fluorescence CDs. The structure and fluorescence properties of these CDs were subsequently characterised. In the bottom-up method, carbon-based small molecules and carbon-rich fragments are used as raw materials. In the hydrothermal, microwave, and ultrasonic methods, polymerisation and carbonisation of the carbon source result in the formation of CDs such as those shown in Figure 2b. Compared with the top-down method, the bottom-up method involves materials that can be obtained from a wider variety of sources. Further, it involves simpler synthesis routes and incurs lower equipment costs; therefore, it is preferred for CD preparation.

### 2.3. Arc Discharge Method

In 2004, Xu [29] inadvertently obtained fluorescent substances during the preparation of pure single-walled CNTs by using a 3 M HNO_3_ solution as the oxidant to achieve oxidation arc discharge. Under alkaline conditions (NaOH, pH = 8),e HNO_3_ was neutralised, yielding a suspension containing single-walled CNTs and other impurities. With subsequent separation, a fluorescent substance was obtained. Gel electrophoresis was used to isolate the purified fluorescent substance, from which electrophoretic bands corresponding to blue-green, yellow, and orange lasers were obtained. It was found that three types of fluorescent substances could be separated via arc discharge. However, this approach tends to produce separated substances that contain significant amounts of impurities that are difficult to separate, leading to low yields.

### 2.4. Laser Erosion Method

Graphite powder and cement have been used to obtain carbon materials via heat treatment [30]. In the presence of a water vapour current, these carbon materials were transformed into carbon particles via laser erosion. The resulting carbon particles were then compounded to form fluorescent particles of various sizes. Subsequently, the fluorescent particles were placed in a 2.6 M HNO_3_ solution for 12 h under backflow conditions, and the resulting uniformly sized carbon particles were decorated with PEG1500 to obtain particles with an average diameter of 5 nm. At an excitation wavelength of 400 nm, the yield of the prepared carbon quantum dots was found to increase from 4% to 10%. Further, Hu [31] used a pulsed laser to illuminate graphite sheets in a polymer solution. The pulse duration was found to affect the resulting particle size and the fluorescence quantum yield (QY). The laser erosion method is advantageous; however, large amounts of the carbon source are required, and the preparation of heterogeneously sized carbon nanoparticles significantly restricts the application of the resulting CDs.

### 2.5. Microwave Method

The microwave method is simple, rapid, and highly sensitive; therefore, it has been widely employed for the preparation of CDs. For example, Qu [32] used citric acid and urea as raw materials to prepare water-soluble CDs. This simple route involves mixing of the reactants in the microwave digestion system and heating at 750 W for 4–5 min to produce a brown CD solution. The desired CDs are then isolated via centrifugal purification. In addition, Zhang [33] used the microwave method to prepare green CDs for the fluorescence probe detection of folate receptor-positive cancer cells. Additionally, He prepared CDs via the carbonisation of active dry yeast through the microwave approach. The average particle size of the resulting CDs was determined to be 3.4 nm, and their maximum emission peak was observed at ~516 nm. Furthermore, Liu [34] used the microwave method (400 W, 30 min) to prepare orange, yellow, and blue CDs that can be used in colour-based cell imaging and illumination, thereby expanding the application scope of CDs. It was also possible to use these CDs for the detection of cell pH levels. Compared with other methods, the synthesis time required by the microwave method is short, the equipment is simple, and the raw materials are inexpensive and widely available. Consequently, this is one of the most popular approaches for preparing CDs.

### 2.6. Hydrothermal Method

The hydrothermal method has numerous advantages, including a simple apparatus, facile operation, low cost, and eco-friendliness. Zhou used ammonium citrate as the carbon source, and, through a hydrothermal reaction at 180 °C for 30 min, obtained the desired CDs possessing applicability in the rapid detection of ochratoxin A in flour and beers. Chandra [35] used citric acid and ammonium dihydrogen phosphate as the raw materials for their hydrothermal reaction. After 4 h of reaction at 180 °C and subsequent centrifugation, the purified CDs were obtained via dialysis. The prepared CDs were then used to measure the Fe^3+^ contents of cancer cells. Further, Long used the hydrothermal method to prepare CDs containing F and N. When these CDs were combined with room-temperature phosphors, they were suitable for use as protective materials for data security. Furthermore, the hydrothermal method has been used to prepare organic-soluble CDs and multicolour fluorescence emission-adjustable CDs. Moreover, Yuan [36] used citric acid and either 2,3-diaminonaphthalene (2,3-DAN) or 1,5-DAN as the raw materials under different reaction times and solvent contents to control the carbonisation process. They found that the emission peaks of the resulting fluorescent substances exhibited a red shift. Fluorescent CDs exhibiting blue, green, yellow, orange, and red colours were also prepared. It has been reported that the luminescence mechanism of multicolour fluorescence emission-adjustable CDs is affected by various factors, e.g., changes in the CD bandgap, the charge transfer on the CD surface, and the sizes of the CD molecules. Overall, the CDs prepared using the hydrothermal approach are widely applicable, and the carbon sources tend to be cheap and readily available. However, the CD luminescence mechanism remains to be elucidated. Further, novel methods are needed to increase the CD QYs.

## 3. Fluorescence Properties of CDs

### 3.1. Synthesis of High-Efficiency N,S-Doped Blue CDs

At present, the quantum efficiency of CDs is low. Thus, increasing the quantum efficiency is the main priority in the CD research field. High-efficiency CDs are prerequisites for composite applications. For the experiment performed in this study, the convenient hydrothermal method was selected. Citrate sodium and cysteine were used as raw materials. Ethylenediamine was used as a passivation agent. High-efficiency N,S-doped blue CDs were synthesised. Quinine sulphate (0.1 M H_2_SO_4_, QY = 56%) was used as a standard reference solution. The quantum efficiency of the CDs was calculated as 68%. The fluorescence of the CDs was examined, along with the effects of the NaCl solution, pH, ultraviolet (UV) light, temperature, and metal ions on the CD fluorescence intensity. All the reagents used in the experiment are presented in Table 1.

The experiment instruments are shown in Table 2.

### 3.2. Synthesis of N,S-Doped Blue CDs

The blue CDs were synthesised using a one-step hydrothermal method via the following steps. First, 0.2 g of L-cysteine and 0.2 g of citrate sodium were weighed and added to a 25-mL beaker. Subsequently, 10 mL of ultrapure water and 500 μL of ethylenediamine (EDA) were added via pipette for 5 min until complete dissolution. This transparent liquid was shifted quickly to a 50-mL hydrothermal reactor. Then, the reactor was kept in an oven at 200 °C for 4 h. After the liquid cooled to room temperature, dialysis was performed for 24 h by using a dialysis bag (Mw = 1000). Thus, purified N,S-doped CDs with an average size of 5 nm were obtained.

### 3.3. Fluorescence Spectra

The excitation and emission spectra, temperature-dependence emission spectra, and fluorescence lifetimes of the samples were measured using X-ray fluorescence (FLS980). The illuminant in the emission and photostability tests was an Xe lamp, and that in the fluorescence lifetime test was a 320 nm laser. Next, 3 mL of the CD solution was added to a cuvette. The slit was set as 1.5 nm. The residence time was 0.05 s. The excitation wavelength was 350 nm. The emission spectra were acquired within the range of 360–750 nm. Additionally, the CD influence factors (pH, metal ions, and NaCl) were tested via the following steps. First, 3 mL of buffer solution with different pH values was prepared. Next, 100 μL of CD solution was added, and the mixture was shaken. The fluorescence intensity test (*λ*_ex_ = 350 nm) was then conducted. To examine the effect of the concentration of the NaCl solution on the CDs, NaCl solutions with concentrations of 0.2, 0.4, 0.6, 0.8, 1.0, and 1.2 M were used; 100 μL of the CD solution was added, followed by mixing for 3 min. Finally, the emission spectra were acquired.

**Figure 3:**
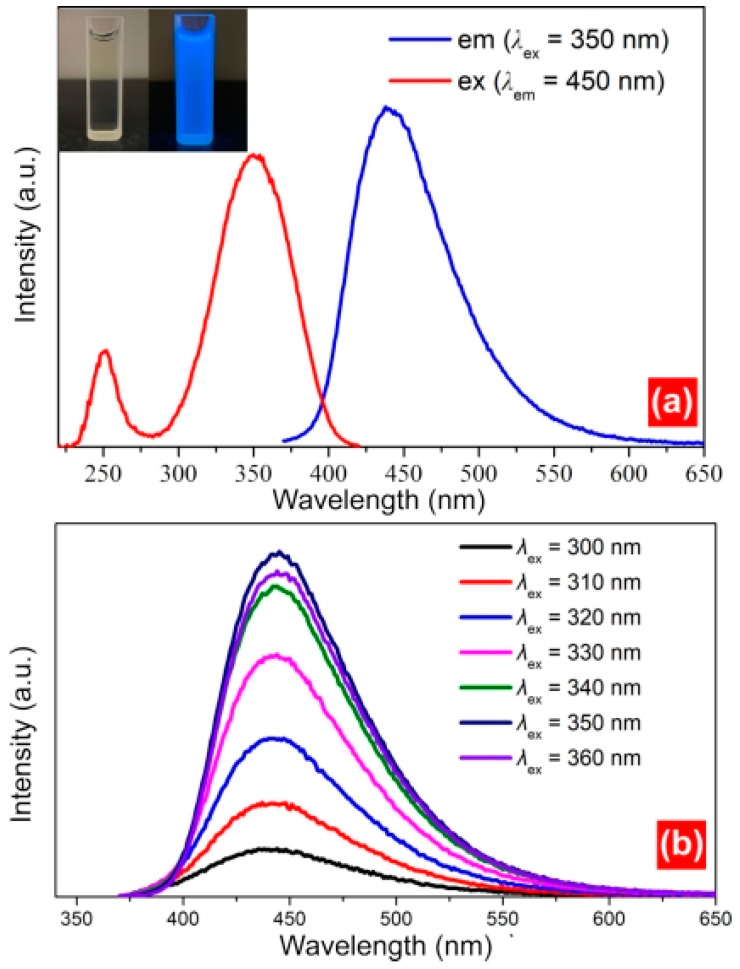
(**a**) Excitation and emission spectra of CDsand (**b**) effect of different excitation wavelengths on the fluorescence intensity of CDs.

The photoluminescence excitation (PLE) spectrum and emission spectrum (PL) are important for measuring the fluorescence properties of CDs. In this study, a steady/transient-state fluorescence analyser (FLS980) was used to analyse the PLE and PL of the samples (Figure 3a). Under natural light, the CD solution was faint yellow and transparent; however, under UV irradiation, the light emitted by the CDs was blue. The optimum excitation wavelength (*λ*_ex_) and strongest emission peak of the CDs were 350 and 450 nm, respectively. Calculations using quinine sulphate (QY = 56%) as a standard reference solution indicated that the QY of the CDs increased to 68%; this QY exceeded those of other CDs, such as S/N-doped CDs (13.71%), full-colour electroluminescent CDs (35%), and BNS CDs (5.44%). The emission behaviour of the samples is also important, as differences in the excitation wavelength can result in a redshift or blueshift of the emission spectrum. The emission behaviour can be divided into excitation wavelength-dependent behaviour and non-excitation wavelength-dependent behaviour. An excitation wavelength ranging from 300 to 360 nm was used to assess the emission behaviour of the CDs. The emission spectrum was analysed at 10 nm intervals within the testing range of the excitation wavelength. However, only one emission peak at 450 nm was observed for each spectrum (Figure 3b). Thus, the emission spectrum did not exhibit a redshift or blue shift across different excitation wavelengths, indicating that the emission behaviour of the CDs did not depend on the excitation wavelength. This is because the size of the aromatic ring on the surface of the CDs was relatively uniform, and the emission locus was close.

**Figure 4:**
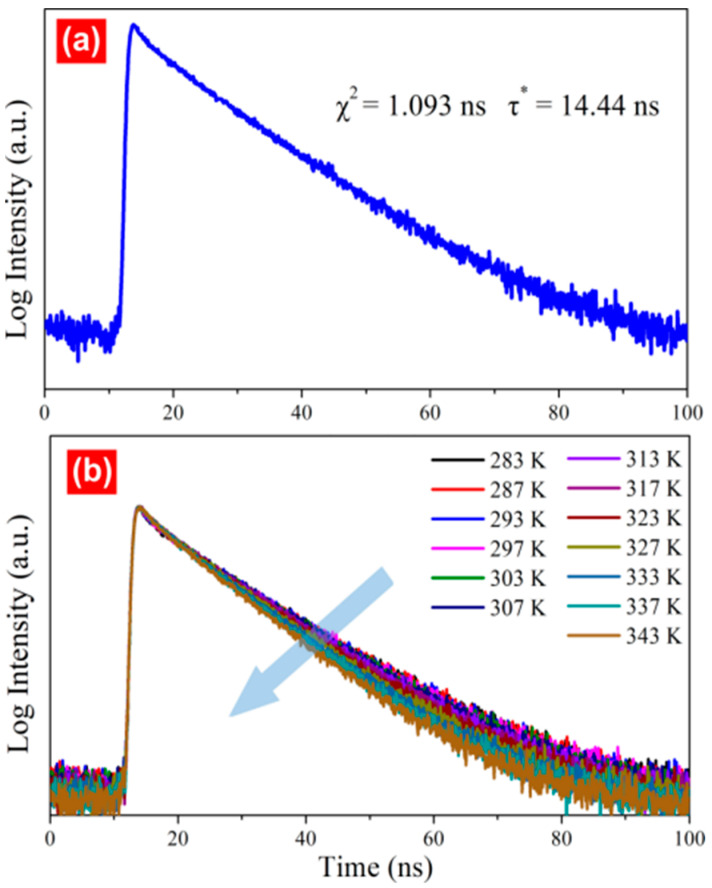
Fitted curve for the fluorescence decay of CDs. (**a**) Everage life of CD (**b**) Temperature-life dependence graph of CD.

The fluorescence lifetime test was performed to analyse the fluorescence decay characteristics of the CDs. The double-exponential decay dynamics function (*χ*^2^ = 1.093) was successfully fitted. At room temperature, the fluorescence lifetimes were 2.48 ns (3.48%) and 14.87 ns (96.52%) (Figure 4a), and the average lifetime of the CDs was 14.44 ns; this short lifespan was consistent with the blue fluorescence decay characteristics. Chen previously reported an average lifetime (12 ns) that was similar to the results of this study. The fluorescence lifetime was also affected by the temperature; an increase in the temperature from 283 to 343 K reduced the fluorescence lifetime from 15.03 to 11.75 ns (Figure 4b). This occurred because of nonradiative relaxation. The blue arrow meas the Non radiative relaxation process.

### 3.4. Analysis of CD Morphology

**Figure 5:**
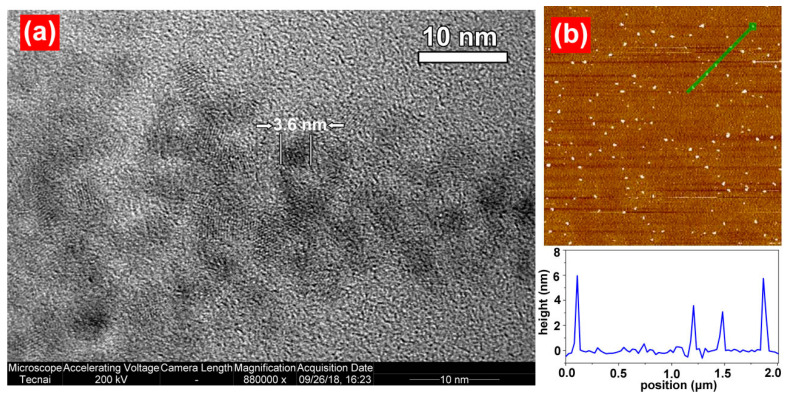
TEM and AFM images of CDs. (**a**) TEM image of CDs (**b**) AFM image of CDs.

The CD size and morphology were examined via TEM, as shown in Figure 5a. The CDs synthesised via the hydrothermal method were spherical and well-dispersed. The average particle size was 3.6 nm. As shown in Figure 5b, these findings were supported by atomic force microscopy (AFM) test results (The green arrow point to the CDs). To measure the electric charges in the CDs, a potential test was performed using a Zetasizer Nano potentiostat. After parallel testing was performed three times, the charge in the CDs was −38 mV. The groups on the CD surface were ionised easily, yielding anions; thus, the negative electricity was strong. These CDs can be used for the detection of positively charged species. Additionally, they can combine with positively charged species, forming composites.

## 4. Carbon-Dot Applications

Given their exceptional fluorescence and biocompatibility, CDs are commonly used in various applications, including drug testing, cell imaging, chemical sensing, and information forgery-proofing. In this section, we explore the use of CDs in four key areas––electro-optical devices, detection of drug ions, biological imaging, and information forgery-proofing.

### 4.1. Electro-Optical Devices

Typically, CDs are prepared in aqueous solutions. Even when prepared using the microwave method, CDs only exhibit fluorescence when dissolved in water. However, CDs must be in the solid state for application to electro-optical devices, and severe aggregation-induced quenching remains a significant challenge in this regard. Tian [37] synthesised controllable-luminescence CDs that emitted light ranging from blue to red. To overcome the issue of aggregation-induced quenching, a microwave-assisted rapid heating method was used to solidify the CDs in a sodium silicate aqueous solution. The solid-state CDs maintained their fluorescence, which allowed the preparation of multicolour light-emitting diode (LED) and WLED lighting devices based on CDs. Zhai [38] synthesised red-, green-, and yellow-fluorescent CDs, which were combined with blue chips to construct WLEDs with adjustable colour temperatures. Wang [39] synthesised red CDs with a quantum efficiency of 53%, which were combined with blue- and green-fluorescent powders to prepare warm white LEDs. These LEDs exhibited excellent overall performance, including a correlation colour temperature of 2875 K, colour rendering index of 97, and luminous efficacy of 31.3 lm/W, representing a significant contribution to the development and application of high-efficiency composite LEDs based on CDs.

### 4.2. Drug and Ion Detection

CDs exhibit excellent fluorescent properties; however, their fluorescence is quenched when they are combined with drugs or ions. This property makes CDs useful for the quantitative detection of drugs and ions.

**Figure 6:**
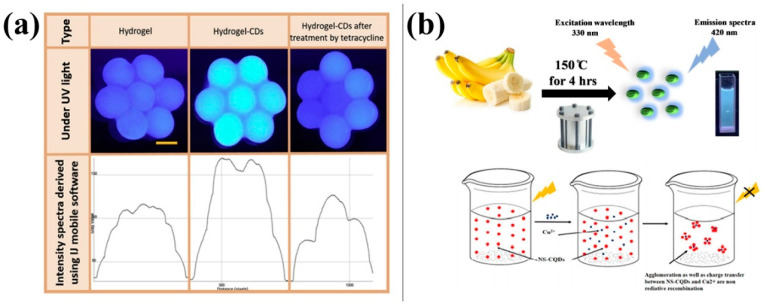
Carbon dots (CDs) for drug and ion detection: (**a**) CD composite hydrogels for tetracycline detection and absorbance [41], (**b**) CDs for copper ion detection [46].

Tetracycline is a broad-spectrum antibiotic that inhibits the growth of multiple Gram-positive and Gram-negative bacteria. It is also effective against rickettsia and trachoma virus. Guo [40] developed a method to produce blue-fluorescent CDs by thermally cracking crab waste material. These CDs have been successfully utilised to detect tetracycline with good detection stability and low detection limits. They can be applied to quantitative detection of tetracycline in most acidic solutions. Furthermore, this method has been shown to be useful for sewage water analysis. Ehtesabi [41] synthesised blue-fluorescent CDs via pyrolysis and characterised them. Upon exposure to tetracycline, the fluorescence emission intensity of the CDs was significantly reduced. To develop a user-friendly tetracycline detection method, the synthesised CDs were encapsulated in a chondrocyte-sodium hydrogel. Figure 6a shows the fluorescence quenching intensity of CDs encapsulated in the hydrogel structure measured in the presence of tetracycline. The encapsulation of CDs in hydrogels enables their use as a tetracycline sensor and as an adsorbent for environmental tetracycline pollutants. The new method is faster and more efficient than traditional tetracycline detection methods. Folic acid is a pteridine derivative that was initially separated from heparin and is abundant in green plants. Tu [42] used citric acid, EDTA, and mesoporous silica (MCM-41) to prepare blue-fluorescent CDs. Their research indicated that folic acid can selectively quench CD fluorescence. Zhang [43] used these CDs to analyse folic acid in human urine samples, and the recovery rates for the samples were between 82.0% and 113.1%. The results indicated the effectiveness of this method for folic-acid analysis.

Heavy-metal ions can cause severe environmental pollution, as they cannot be easily dispersed and tend to bioaccumulate in the food chain and ultimately in the human body, resulting in metal poisoning. Therefore, detecting their presence in the environment and food is crucial. Qin [44] used microwave irradiation to prepare CDs that served as effective fluorescence sensing probes that enable sensitive and selective detection of Hg^2+^ with a detection limit as low as 0.5 nM. Through a hydrothermal method with coconut juice as the raw material, Murugesan [45] prepared fluorescent CDs that could detect Ag ions in aqueous solutions with a detection limit of 0.26 nM. These CDs can be used as a material for fluorescent probes for environmental protection against metal ions. Figure 6 shows the fluorescence of CDs prepared by Chaudhary [46]; these CDs could measure the concentration of Cu ions with a detection range of 1–800 μg/mL and a detection limit of 0.3 μg/mL.

### 4.3. Biological Imaging

Compared with traditional fluorescent materials, CDs exhibit low toxicity and high biocompatibility, thereby reducing the risk of side effects when they enter the body [47,48]. Additionally, most CDs are water-soluble, which simplifies sample preparation by avoiding the need for complex pre-treatment procedures. Moreover, the fluorescence signal of CDs is stable and is less likely to be affected by the complex biological environment. Owing to these significant advantages, CDs are increasingly being used in biological imaging.

CDs with efficient excitation and ionisation emissions in the deep-red/NIR spectral range are important for bioimaging applications. Liu developed a simple and effective method to significantly enhance the absorption and emission of CDs in the deep-red/NIR spectral range by suppressing nonradiative charge recombination via deprotonation of the CD surface. Owing to an enhanced deprotonation ability and increased viscosity, the NIR emissions of CDs in N,N-dimethylformamide and glycerol at −20 °C exhibited 50- and 70-fold increases, respectively, compared with those of CDs in aqueous solutions at room temperature. Given the adjustable NIR fluorescence intensity of CDs, multilevel data encryption in the NIR region was achieved by controlling the humidity and temperature of CD-ink-stamped paper.

**Figure 7:**
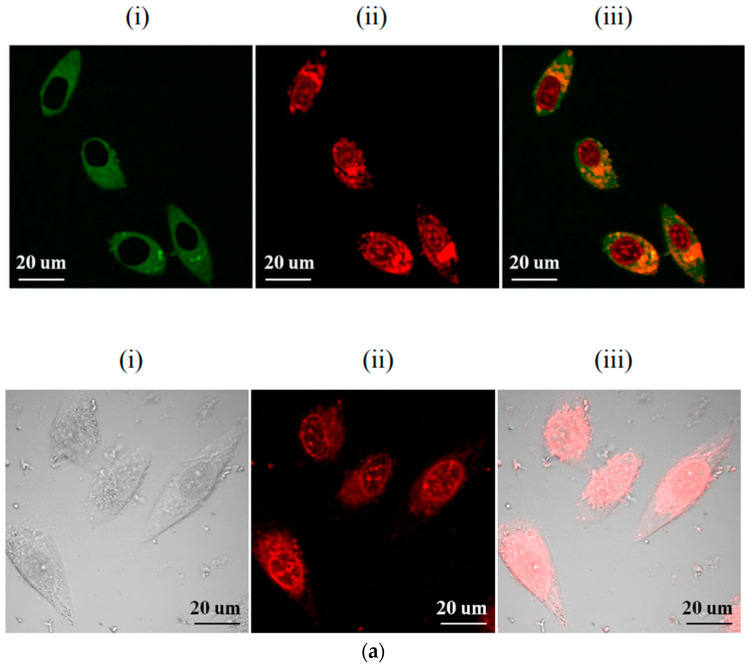

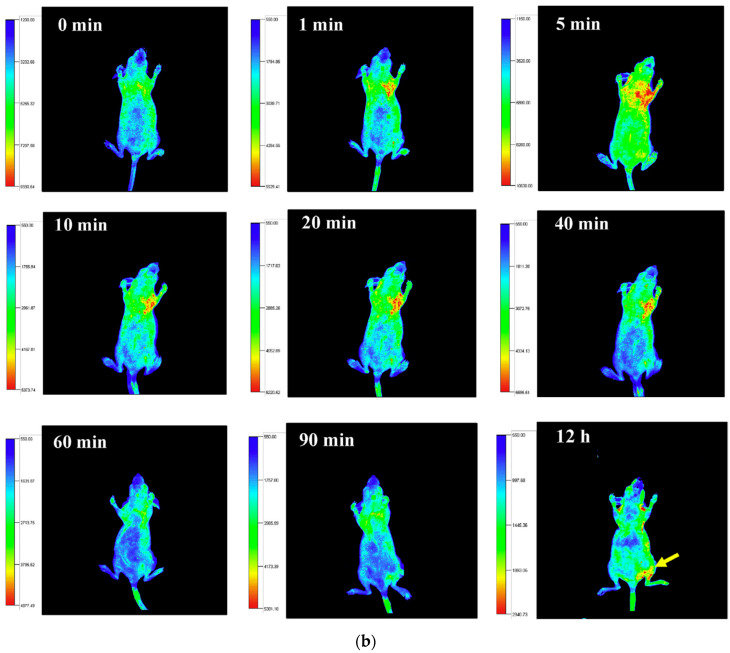
Carbon dots (CDs) for biological imaging: (**a**) CDs for cell imaging [49], (**b**) CDs for in vivo imaging of mice [51].

Gong et al. [49] used amylacea, EDA, and strong phosphoric acid (SPA) to prepare a cavity CD with P and N double doping. The CD prepared by amylacea, EDA, and strong phosphoric acid are shown in Figure 7a(i), (ii) and (iii) respectively. As shown in Figure 7a, these CDs can serve as nanocarriers for anticancer drugs such as doxorubicin, inhibiting tumour growth while allowing fluorescence imaging. Chen [50] synthesised a series of hydrophobically modified CDs with low cytotoxicity via a ring-opening reaction. These CDs exhibited reduced cytotoxicity and improved serum survivability. Moreover, they enable dual-channel imaging and can be used to track the transfer of DNA in cells. Cell-uptake experiments confirmed that these CDs have excellent serum survivability and a strong structure–activity relationship. Yang [51] prepared F-doped CDs using a simple and eco-friendly one-step microwave-assisted carbonation method. As shown in Figure 7b, these CDs emitted red fluorescence, and the researchers suggested a possible mechanism for the emission redshift caused by F doping. These CDs can be used as optical nanoprobes for biological imaging within the human body. The results indicate that F-doped CDs have potential for tumour biological imaging and diagnosis. In summary, research on CDs for biological imaging is constantly advancing, and CDs are expected to be increasingly used as internal fluorescence probes.

### 4.4. Information Anti-Counterfeiting and Encryption

Fluorescent anti-counterfeiting refers to security patterns or characters printed using fluorescent ink that are invisible under normal lighting conditions. However, when exposed to UV radiation, these patterns become visible. The recoverable fluorescence characteristics of CDs can be used for information anti-counterfeiting and encryption.

**Figure 8:**
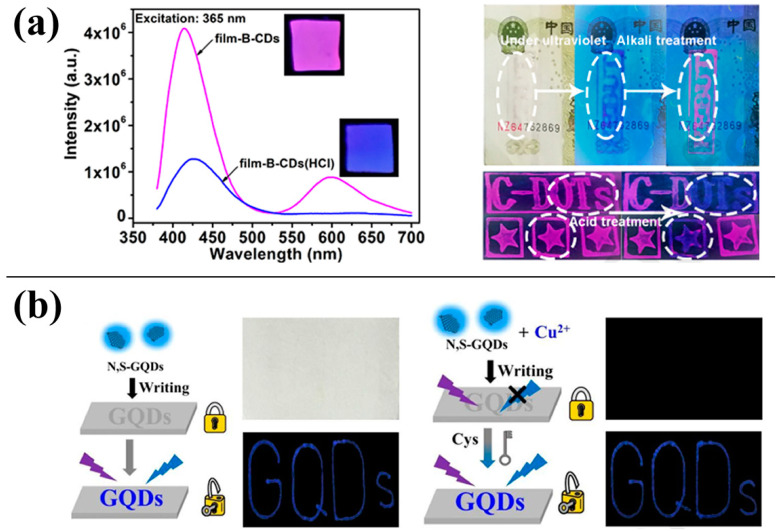
Carbon dots (CDs) used for information security: (**a**) double-mode information security effect of CDs [53], (**b**) encryption and decryption function of CDs [54].

Guo [52] prepared bright green-yellow-emitting CDs via a hydrothermal method using 2-hydroxyphenyl boric acid and EDTA as a solvent. These CDs can be used to measure the concentration of Cr_2_O_7_^2−^ in water. Furthermore, they can be used as fluorescent ink, emitting bright green light under UV radiation. Yuan [53] synthesised red-fluorescent CDs that were sensitive to acid and attached them to a polyvinyl alcohol (PVA) membrane. As shown in Figure 8a, the CDs without hydrochloric acid treatment exhibited red fluorescence under UV irradiation. After treatment with hydrochloric acid, the fluorescence was blue, facilitating a dual-mode information anti-counterfeiting application. Zhao [54] synthesised three types of CDs with red, green, and blue fluorescence. The blue-fluorescent CDs were used to prepare a fluorescent ink, as shown in Figure 8b. When this ink was applied to paper, the blue fluorescence was visible under UV radiation. However, it disappeared when the ink was sprayed with a Cu^2+^ solution, providing encryption. The fluorescence can be recovered upon spraying with a cysteine solution, providing decryption. This method based on fluorescent CDs is a promising new approach to anti-counterfeiting and encryption.

### 4.5. Introduction to Danio rerio Model

The zebrafish (*Danio rerio*) is a model organism with several advantages over tradition animal models, for example, small size, highly transparent bodies, high reproduction rates, and high hatchability. The sequencing of the *Danio rerio* genome has revealed considerable homology with the genomes of mammals. Of the 26000 genes of the zebrafish, 70% are similar to those of humans, including genes encoding cytokines and histocompatibility system molecules, which are immune-reaction regulatory factors. Additionally, 84% of known human disease-causing genes correspond to those of the zebrafish [55,56]. Owing to these similarities, the related mechanisms of zebrafish and the pathogenic hosts are similar to those of humans [57,58]. The *Danio rerio* embryo is considered a type of in vitro animal model; accordingly, simple cell or tissue culture experiments can be used to validate the results of animal (e.g., rodent) experiments.

**Figure 9:**
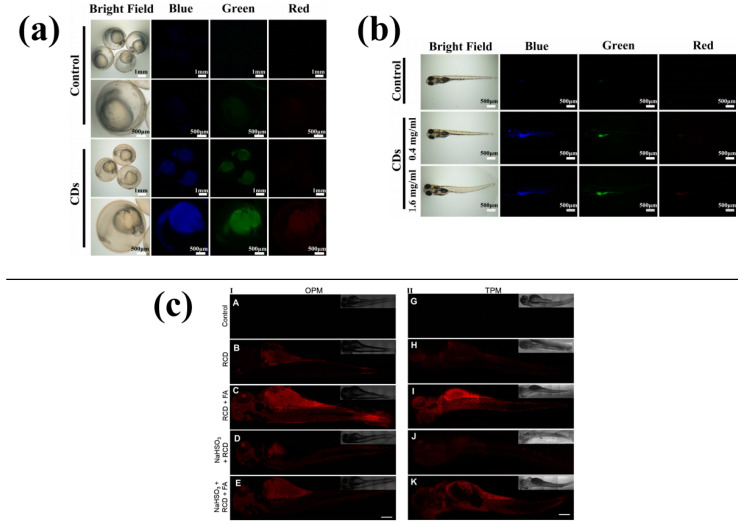
Imaging of zebrafish: (**a**) CDs for imaging zebrafish embryos [59], (**b**) CDs for imaging adult zebrafish [59], (**c**) CDs for imaging formaldehyde in zebrafish [60].

Because of their transparency and short reproductive cycle, zebrafish embryos are particularly well-suited for fluorescence imaging. Wei [59] synthesised a type of blue CDs, and the results showed that different concentrations of CDs had low toxicity to zebrafish embryo development. The nervous and circulatory systems of zebrafish embryos developed normally in the presence of CDs. Figure 9a,b show that the CDs possess great florescent stability and biocompatibility, making them suitable for biological imaging in zebrafish. The CDs can enter the zebrafish embryo through the chorion or oral cavity. Wang [60] prepared red CDs as an unmarked nanoprobe, and the addition of formaldehyde increased the fluorescence of the CDs in zebrafish, allowing for selective testing of formaldehyde in living cells and zebrafish, which is shown in Figure 9c (The different addition of formaldehyde in A–K is 10, 20, 30, 40, 50, 60, 70, 80, 90, 100, 110 mol/L).

## 5. Conclusions

To summarise, CDs are a type of florescent nanomaterial that has garnered a lot of interest due to their excellent fluorescence properties and biocompatibility. Despite the fact that the structure, luminescence mechanism and quenching mechanism of CDs are still not completely understood, research on CDs continues. The research on CD composite methods is relatively mature, and breakthroughs have been made in their application in drug and ion detection, electro-optical devices and biological imaging. The synthesis and application of long-wavelength fluorescent CDs are also widely researched. Additionally, CDs are a new type of florescent nanomaterial that possess many advantages, such as low toxicity, good biocompatibility, unique optical properties and non-bleaching fluorescence, which overcome some of the disadvantages of traditional florescent materials. With the development of society, the demand for functionalised composites is increasing, and CDs are playing an important role. Synthesis methods for CDs have increased along with the development of CDs. The physicochemical properties of CDs, such as particle size, fluorescence intensity, stability, and water-solubility, can be significantly affected by the synthesis method used, as well as reaction times, temperatures, and pH values. Therefore, controlling these parameters during the synthesis process is crucial for obtaining CDs with desired properties for specific applications.

## Figures and Tables

**Table 1:** Chemical reagents used in the experiments.

Reagent	Purity	Manufacturer
Citrate sodium	Analytical pure	Aladdin Reagent Co., Ltd., Shanghai, China
L-Cysteine	Analytical pure	GHTECH Co., Ltd., Guangzhou, China
EDA	Analytical pure	Aladdin Reagent Co., Ltd., Shanghai, China
Citric acid	Analytical pure	DaMao chemical reagent factory, Tianjin, China
Citrate sodium	Analytical pure	Aladdin Reagent Co., Ltd., Shanghai, China

**Table 2:** Experimental instruments.

Instruments	Model	Manufacturer
Steady/transient state X-ray fluorescence (XRF) spectrometer	FLS980	Edinburgh Instruments company, Edinburgh, Britain
Fourier-transform infrared (FT-IR) spectroscope	Spectrum Two FT-IR	Thermo Fisher Scientific technology company, Waltham, MA, USA
UV-visible-near infrared light spectrophotometer	UV-5500PC	Shimadzu corporation, Kyoto, Japan
Transmission electron microscope	Tecnai G2 F20	Oxford instrument technology Co., Ltd., Shanghai, China
Constant magnetic stirring	85-2	Thermo Fisher Scientific technology company, Waltham, MA, USA
Table-top high-speed centrifuge	TG16-WS	Xiangyi laboratory Instrument Development Co., Ltd., Xiangtan, China
Collector type constant-temperature heating magnetic stirrer	DF-101S	Yuhua instrument Co., Ltd., Gongyi, China
Electronic analytical balance	AX124 ZH/E	OHAUS instrument Co., Ltd., Newark, NI, USA
Camera obscura UV analyser	ZF-20D	Yuhua instrument Co., Ltd., Gongyi, China
Electric blast drying oven	DHG-9145A	Yiheng scientific instrument Co., Ltd., Shanghai, China
